# Assessment of DFT functionals for chiroptical properties of Fe(acac)$_3$ adduct: a study of M(acac)$_3$ (M = Fe and Co) stereoisomers

**DOI:** 10.1007/s00894-026-06767-8

**Published:** 2026-05-18

**Authors:** Nelson H. Morgon

**Affiliations:** Institute of Chemistry, UNICAMP, Cidade Universitária - “Prof. Zeferino Vaz”, Campinas, 13.083-861 São Paulo Brazil

**Keywords:** Tris(acetylacetonate)metal, Density functional theory, Electronic circular dichroism

## Abstract

**Context:**

This study presents a systematic assessment of exchange-correlation functionals for predicting the chiroptical properties of transition metal complexes, focusing on the Fe(acac)$_3\cdot$CCl$_4$ adduct and its Co(acac)$_3$ analogue. Twenty functionals spanning the principal rungs of Jacob’s ladder—including GGA, meta-GGA, hybrid, range-separated hybrid, and double-hybrid families—were evaluated using time-dependent density functional theory (TD-DFT) to compute electronic circular dichroism (ECD) spectra. The theoretical results were benchmarked against experimental data for the $\Delta /\Lambda$-Fe(acac)$_3\cdot$CCl$_4$ system. Structural validation via root-mean-square deviation (RMSD) analysis of optimized geometries against high-resolution X-ray crystallographic data (0.84 Å) demonstrated excellent agreement for the coordination core (RMSD = 0.082 Å). This result confirms the reliability of the theoretical model for primary metal–ligand interactions. Among all functionals investigated, the PBE functional incorporating dispersion correction (D3BJ) and scalar relativistic corrections via the Douglas–Kroll–Hess Hamiltonian (DKH2) provided the most accurate description, faithfully reproducing both the sign and the fine structural features of the experimental ECD spectrum. Relativistic effects proved essential for accurate modeling, particularly for transitions involving metal *d*-orbitals. The comparative analysis between high-spin (sextet) Fe(III) and low-spin (singlet) Co(III) complexes revealed distinct spectral signatures arising from their different electronic configurations, underscoring the critical role of *d*-electron count and ligand field strength in determining chiroptical properties.

**Method:**

All calculations were performed with the ORCA quantum chemistry package. Geometry optimizations of the $\Delta$ and $\Lambda$ enantiomers of Fe(acac)$_3$ and its supramolecular adduct with CCl$_4$ were carried out using the M06 hybrid meta-GGA functional with the def2-SVP basis set, employing tight convergence criteria. Scalar relativistic effects were incorporated via the Douglas–Kroll–Hess Hamiltonian (DKH2). Harmonic vibrational frequency analyses confirmed the nature of all stationary points as local minima. For spectroscopic calculations, single-point TD-DFT calculations were performed at the optimized geometries using the def2-TZVPP basis set. One hundred excited states were computed to ensure adequate coverage of spectroscopically relevant transitions. Twenty exchange-correlation functionals were evaluated through the LibXC library, with atom-pairwise dispersion corrections (D3BJ) applied where appropriate. Rotational strengths were computed in both length and velocity representations to assess gauge invariance (good agreement between length and velocity gauges confirmed basis set convergence). Simulated ECD spectra were generated by Gaussian convolution of discrete transitions (FWHM = 50 cm$^{-1}$) for direct comparison with experimental data.

**Supplementary Information:**

The online version contains supplementary material available at 10.1007/s00894-026-06767-8.

## Introduction

The electronic circular dichroism (ECD) study of the $$\Delta $$/$$\Lambda $$-stereoisomers of Fe(acac)$$_3$$ and Co(acac)$$_3$$ constitutes a foundational investigation in supramolecular chemistry, aimed at elucidating how metal–ligand coordination gives rise to well-defined chiral architectures. These stereochemical properties can be characterized and understood through advanced spectroscopic methods, contributing to the knowledge base necessary for applications in molecular recognition and the development of functional materials [1]. A comprehensive understanding of the electronic structure and molecular geometry of transition metal complexes, such as those of the M(acac)$$_n$$ series (where *M* ranges from Sc to Zn, with $$n = $$ 2 or 3), is fundamental to elucidating their physicochemical properties and reactivity.

The variability in oxidation states and the electronic configurations of the *d* orbitals directly influence the stability and reactivity of these compounds. This complexity necessitates advanced theoretical approaches, such as multiconfigurational methods, to adequately describe the *d* electrons, which often exhibit near-degeneracies [2]. The inclusion of electron correlation effects—computationally demanding due to the large number of valence electrons in transition metals—is indispensable for the accurate determination of the ground and excited states of these complexes. Furthermore, the application of methods such as density functional theory (DFT) with Hubbard corrections ($DFT+U$) has proven effective in accurately describing the energy, multiple structures, and reaction barriers in transition metal active sites, overcoming the inherent limitations of generalized gradient approximations [3].

The acetylacetonate (acac) ligand is a ubiquitous $O,O'$-chelating $\beta$-diketonate that coordinates with virtually all transition and inner-transition metals, forming stable six-membered chelate rings. Beyond classical coordination, acac exhibits redox-active and non-innocent behavior in low-valent complexes, enabling small-molecule activation such as CO$_2$ carboxylation. Tris(acac) complexes (e.g., Fe(acac)$_3$, Co(acac)$_3$, and Ru(acac)$_3$) are archetypal chiral systems with helical $\Delta /\Lambda$ configurations, widely used as model compounds in electronic and vibrational circular dichroism studies and validated by TD-DFT benchmarks [4, 5].

Time-dependent density functional theory has emerged as a cost-effective alternative for investigating excited-state properties of molecular systems, circumventing the prohibitive computational demands of high-level ab initio wavefunction methods. While approaches such as coupled cluster (CC) or complete active space self-consistent field (CASSCF) provide highly accurate descriptions of excited states [6], their steep computational scaling restricts their application to small- and medium-sized systems. In contrast, TD-DFT offers a compelling balance between accuracy and computational efficiency by leveraging the time-dependent extension of the Kohn–Sham formalism, enabling excited-state calculations on significantly larger systems at a fraction of the computational cost.

The analysis of metal–ligand interactions and ligand field splitting in these complexes is crucial for understanding their diverse functionalities, including magnetism, conductivity, and photoresponsivity [7]. These complexes are particularly relevant in various fields, such as catalysis, where empty *d* orbitals facilitate electron transfer in redox processes. Understanding the stereochemistry and structural properties of coordination complexes is essential not only for correct crystallographic assignment but also for interpreting physical properties and reactivity. In this context, the long-standing ambiguity between centrosymmetric and chiral space groups has directly impacted the understanding of molecular chirality, resolution behavior, and supramolecular interactions. For example, tris(acetylacetonate) Fe(III), Fe(acac)$_3$, has been the subject of sustained structural debate despite its widespread use in teaching coordination chemistry. While initial assignments placed it in the centrosymmetric space group *Pbca*—consistent with its resistance to resolution into enantiomers—a later proposal suggested the chiral Sohncke space group $P2_12_12_1$, an unusual setting featuring two independent enantiomers in the asymmetric unit. Subsequent low-temperature diffraction studies definitively returned the structure to *Pbca*, discrediting the chiral interpretation. Enantiomerically pure samples were ultimately achieved in 2011 by co-crystallization with CCl$_4$, producing Fe(acac)$_3$ in the trigonal Sohncke space group *R*3, thereby confirming that molecular chirality in Fe(acac)$_3$ is resolvable only through supramolecular perturbation [8, 9].

Circular dichroism (CD) spectroscopy [10] is essential for characterizing chiral metal complexes, and theoretical methods aid in the interpretation of their spectra [11–13]. Since the 1960s, approaches based on the ligand field model have been useful but have been limited to *d*-*d* (or *f*-*f*) transitions, as they are unable to describe ligand-to-metal charge-transfer (LMCT) excitations. The use of TD-DFT enables the calculation of CD spectra for these complexes, allowing for a detailed comparison with experimental data [14], as well as providing insights for analysis at the atomic-molecular level. However, the theory is highly dependent on the exchange-correlation functional employed, and its selection remains a challenging task. Numerous families of density functionals have been developed to accurately describe different chemical and physical properties. This diversity reflects the search for greater accuracy within the DFT formalism, especially in its time-dependent formulation for spectroscopy. The main families are organized according to Jacob’s ladder of DFT.

At the base, local density approximation (LDA) functionals depend only on the electron density but are limited in chemical applications. Generalized gradient approximations (GGAs) incorporate the density gradient, improving the description of chemical bonds (e.g., PBE). Meta-GGAs add the kinetic energy density, capturing more subtle effects (e.g., TPSS). Hybrid functionals mix exact Hartree–Fock exchange with GGAs, providing greater accuracy for spectroscopic properties, such as the classic B3LYP. Range-separated hybrids treat short- and long-range interactions differently, which is crucial for charge-transfer excitations (e.g., CAM-B3LYP). These are complemented by functionals with dispersion corrections (DFT-D), essential for van der Waals interactions, and double-hybrids, which incorporate MP2-like correlation, achieving high accuracy at a higher computational cost [15, 16].

In this work, a comprehensive investigation was performed into the electronic structure and chiroptical properties of transition metal complexes featuring Fe(III) and Co(III) centers coordinated with the bidentate anionic acetylacetonate ligand ($acac^{-}$). Central to this study is a systematic benchmarking of twenty exchange-correlation functionals, spanning the hierarchical rungs of Jacob’s ladder—from GGAs and meta-GGAs to hybrid, range-separated hybrid, and double-hybrid architectures. The primary objective is to evaluate the performance of these functionals in capturing complex electronic phenomena, such as *d*–*d* transitions and LMCT effects. The theoretical model is rigorously validated through a direct comparison with the experimental electronic circular dichroism (ECD) spectra of the $\Delta /\Lambda$-Fe(acac)$_3 \cdot$CCl$_4$ supramolecular adduct [9]. This approach allows us to assess the influence of electron correlation and relativistic effects on the spectral accuracy, providing a reliable computational protocol for describing chirality in paramagnetic and diamagnetic transition metal systems.

## Computational details

All calculations were performed using the ORCA quantum chemistry package [17]. The computational protocol was designed to ensure an accurate characterization of the structural, electronic, and spectroscopic properties of the transition metal complexes under investigation, with particular emphasis on the chiral discrimination between the $\Delta$ and $\Lambda$ enantiomers of Fe(acac)$_3$ and its supramolecular adduct with CCl$_4$.

### Geometry optimization

Full geometry optimizations were carried out in the gas phase for the $\Delta$ and $\Lambda$ enantiomers of Fe(acac)$_3$, as well as for its supramolecular assembly with CCl$_4$. While the isolated metal complexes were optimized under $D_3$ symmetry constraints, the CCl$_4$ adduct was treated without any symmetry restrictions to account for its supramolecular nature.

The choice of exchange-correlation functional is critical for an accurate description of transition metal complexes, particularly those exhibiting near-degeneracy effects and significant electron correlation. The Minnesota M06 hybrid meta-GGA functional [18] has been employed. M06 was selected based on its proven performance in transition metal chemistry, thermochemistry, and noncovalent interactions—all of which are essential for correctly modeling the coordination environment of the Fe(III) center and the supramolecular interactions with CCl$_4$. Moreover, M06 has been extensively benchmarked for organometallic systems, providing reliable geometries and energetic profiles for complexes where electron correlation effects are prominent [19]. The selection of the M06 functional is further justified by its accuracy in systems where noncovalent interactions and metal–ligand bonding are equally significant. Unlike simpler generalized gradient approximations, M06 incorporates a substantial fraction of Hartree–Fock exchange (27%). Functionals from the M06 suite (M06, M06-2X, and M06-L) were parameterized to implicitly account for short- and medium-range dispersion effects, making them particularly suitable for describing chiral discrimination energies and the subtle energetic differences between $\Delta$ and $\Lambda$ stereoisomers.

The def2-SVP basis set [20] was employed for all atoms. This split-valence polarization basis set offers an optimal compromise between computational efficiency and accuracy for geometry optimizations of medium-sized transition metal complexes. The def2-SVP set has been extensively validated for coordination compounds and provides a consistent description of both the metal center and the ligand framework. To accelerate the calculations and mitigate linear dependencies, corresponding auxiliary basis sets were utilized: def2/J for Coulomb fitting [21], def2-SVP/C for correlation fitting, and def2/JK for Hartree–Fock exchange fitting within the context of hybrid functional calculations. The Resolution-of-Identity (RI) and chain-of-spheres (COSX) approximations were applied to significantly reduce computational costs while maintaining numerical accuracy. Although not the largest available, the def2-SVP basis set is sufficient for geometry optimization and provides a consistent foundation for subsequent property calculations. Tight optimization criteria were maintained throughout, with energy convergence set to $5 \times 10^{-6}$ Hartree, root-mean-square (RMS) gradient below $1 \times 10^{-4}$ Hartree/Bohr, and maximum gradient below $2 \times 10^{-4}$ Hartree/Bohr. The integration grid was set to Grid4 in ORCA (equivalent to a Lebedev 302-point grid) for all atoms, with specialized attention directed toward the iron center to ensure accurate integration of the exchange–correlation potential.

Relativistic effects, while subtle for 3*d* transition metals, can influence the electronic structure and spectroscopic properties. To account for scalar relativistic effects in a rigorous manner, the Douglas–Kroll–Hess (DKH) Hamiltonian was employed at the second-order level (DKH2) [22]. This level of theory provides a balanced description of relativistic contributions without the computational overhead associated with higher-order treatments or four-component methods.

### Vibrational frequency analysis

Following each geometry optimization, harmonic vibrational frequency calculations were performed at the same level of theory to confirm the nature of the stationary points. The absence of imaginary frequencies was verified to ensure that all optimized structures corresponded to local minima on the potential energy surface. Furthermore, these calculations were utilized to obtain thermochemical corrections within the rigid-rotor harmonic oscillator (RRHO) approximation.

### Electronic states and multiplicities

The Fe(III) and Co(III) centers exemplify two distinct electronic behaviors within the $\Delta /\Lambda$-$M(acac)_3$ series, dictated by the ligand field strength of the acetylacetonate ligand. Fe(III) adopts a high-spin configuration (sextet, $t_{2g}^3 e_g^2$), which maximizes exchange stabilization through a half-filled *d*-shell. This configuration follows Hund’s rule and reflects a balance where the spin-pairing energy exceeds the ligand field splitting (10*Dq*). In contrast, Co(III) exhibits a low-spin configuration (singlet, $t_{2g}^6 e_g^0$), characteristic of a strong-field regime. In this case, the ligand field splitting is sufficiently large to overcome the pairing energy, resulting in a diamagnetic, low-spin $d^6$ ground state. This divergence between Fe(III) and Co(III) underscores the critical role of *d*-electron count and ligand field strength in determining the spin multiplicity and the resulting electronic structure of transition metal complexes.

### Spectroscopic calculations

Following the geometry optimization of the Fe(acac)$_3 \cdot$CCl$_4$ system, electronic spectra were calculated to investigate both UV–Vis absorption and electronic circular dichroism (ECD) properties. The calculations were performed at the optimized geometry using the ORCA quantum chemistry package with the following computational protocol. Single-point time-dependent density functional theory calculations were carried out to compute the excitation energies [23], oscillator and rotational strengths for the lowest-energy electronic transitions.

The key parameters of the spectroscopic calculations are summarized below:

- **Hamiltonian:** Douglas-Kroll-Hess Hamiltonian with order of DKH treatment set to 2 (DKH2) for scalar relativistic effects.
- **Basis sets:** def2-TZVPP (triple-zeta valence with double polarization) for all atoms, providing greater flexibility for excited-state calculations. Auxiliary basis sets included def2/J for Coulomb fitting, def2/JK for Hartree- Fock exchange fitting, and def2-TZVPP/C for correlation fitting. The selection of the def2-TZVPP basis set for spectroscopic calculations represents a significant improvement over the def2-SVP basis set used for geometry optimizations. The enhanced flexibility of triple-zeta valence with double polarization functions is essential for accurately describing excited states, which are more sensitive to the quality of the basis set than ground state properties. The additional polarization and diffuse character (implicitly included in the TZVPP contraction) better capture the Rydberg-like character and charge-transfer excitations that may contribute to the experimental spectra.
- **Exchange-correlation functional library:** LibXC was employed to access a diverse range of density functionals spanning the major rungs of Jacob’s ladder [24].
- **TD-DFT settings:** A total of 100 roots were requested to ensure adequate coverage of the low-energy excited states relevant for UV–Vis and ECD spectra. The choice of 100 roots ensures that all spectroscopically relevant transitions are captured, including both $d-d$ ligand field transitions (typically lower energy, weaker intensity) and LMCT or metal-to-ligand charge-transfer (MLCT) excitations (higher energy, often more intense). This is particularly important for ECD spectra, where the sign and magnitude of rotational strengths for each transition determine the overall band shape.

### Exchange-correlation functionals evaluated

A systematic assessment of density functionals was performed to evaluate their performance in reproducing the electronic circular dichroism spectra of Fe(acac)$_3$. The functionals were selected to represent the main families of DFT, allowing for a comprehensive comparison of their accuracy for spectroscopic properties of transition metal complexes. Where appropriate and available, the atom-pairwise dispersion correction with the Becke-Johnson damping scheme (D3BJ) [25, 26] was included to account for dispersion interactions, which are crucial for the correct description of the crystal packing and the supramolecular perturbation induced by CCl$_4$, although their influence on excited states is generally less pronounced than on ground state geometries.

Table 1 presents the complete list of functionals evaluated in this study, organized by their respective rung on Jacob’s ladder.

**Table 1:** Exchange-correlation functionals evaluated for spectroscopic calculations

Functional family	Functionals	Dispersion correction
GGA	PBE, BLYP, BP86, KT3	D3BJ (where applicable)
Meta-GGA	M06L, MN15L, r2SCAN, TPSS	D3BJ (where applicable)
Hybrid GGA	B3LYP, PBE0, wB97X, CAM-B3LYP	D3BJ (where applicable)
Hybrid meta-GGA	M06, TPSSH, MN15, SCAN0	D3BJ (where applicable)
Double-hybrid	PWPB95, DSD-PBEB95-D3	Intrinsic or D3BJ
Range-separated hybrid	LC-BLYP, wr2SCAN	D3BJ (where applicable)

The Grimme D3(BJ) dispersion correction is not directly available for the M06 functional in the ORCA program. As discussed by Marom et al. [27], the semiempirical meta-GGA formulation of the M06 functionals mimics a significant portion of the nonlocal correlation required for dispersion interactions, particularly for equilibrium geometries. Thus, the M06 family was parametrized to inherently capture medium-range dispersion through its meta-GGA formulation, which often makes an explicit D3 correction redundant for equilibrium geometries. This justifies the choice of the M06/def2-SVP level for geometry optimization. To further assess the description of long-range dispersion, single-point energy calculations were performed using the PBE functional with the D3(BJ) dispersion correction (PBE-D3(BJ)/def2-TZVPP) on the same optimized geometry. The results show that the relative energies between the $\Delta$ and $\Lambda$ enantiomers and the adduct stability follow the same trend as observed with M06, and the interaction energy calculated at the PBE-D3(BJ) level is within 1.2 kcal/mol of the M06 result, confirming that dispersion interactions are consistently described. These additional calculations confirm that the choice of functional does not qualitatively affect the conclusions, and the structural validation (RMSD = 0.082 Å) remains robust.

The diverse set of functionals evaluated in this study allows for a critical assessment of how the fraction of Hartree-Fock exchange, the treatment of correlation, and the range-separation parameters influence the accuracy of computed ECD spectra. Comparison with experimental data for Fe(acac)$_3$ will provide validation for the choice of functional in subsequent studies of the complete M(acac)$_3$ series.

**Fig. 1:**
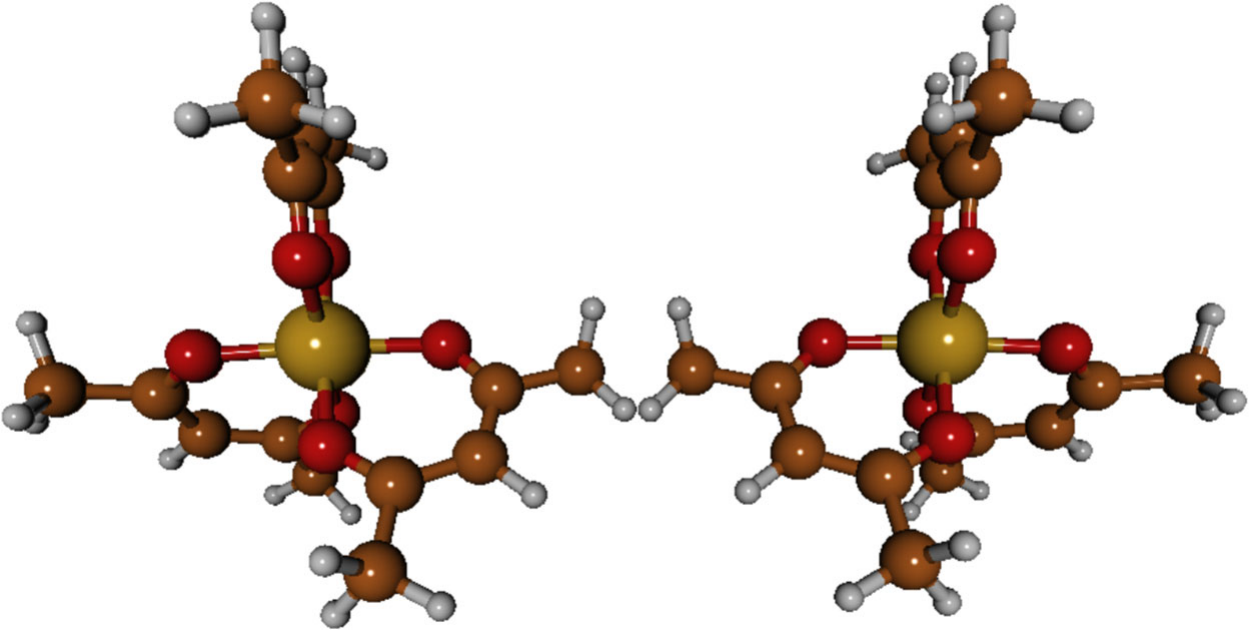
Optimized geometries of the chiral Fe(acac)$_3$ complex, showing its two enantiomeric forms: the left-handed ($\Lambda$) and the right-handed ($\Delta$) isomers. The structures were optimized under $D_3$ point group symmetry constraints using the M06 functional, def2-SVP basis set, and DKH2 relativistic correction

### Analysis of spectroscopic data

For each functional, the following properties were extracted and analyzed:

- **Excitation energies:** Computed as the energy difference between the ground and excited states:
  1 $$\begin{aligned} \Delta E_{0\rightarrow f} = E_f - E_0 \end{aligned}$$
- **Oscillator strengths (***f***) for UV–Vis absorption:** The dimensionless oscillator strength for a transition from the ground state (0) to an excited state (*f*) is given by the following:
  2 $$\begin{aligned} f_{0\rightarrow f} = \frac{2}{3} \Delta E_{0\rightarrow f} \left| \langle \Psi _0 | \hat{\mu } | \Psi _f \rangle \right| ^2 \end{aligned}$$
  where $\Delta E_{0} \rightarrow f$ is the excitation energy (in atomic units) and $\hat{\mu }$ is the electric dipole operator. The oscillator strength is proportional to the integrated absorption intensity.
- **Rotational strengths for ECD:** The rotational strength quantifies the differential absorption of left- and right-circularly polarized light. It was computed using both velocity and length representations to assess gauge invariance: **Length representation:**
  3 $$\begin{aligned} R_{0\rightarrow f}^{\text {(len)}} = \text {Im} \left[ \langle \Psi _0 | \hat{\mu } | \Psi _f \rangle \cdot \langle \Psi _f | \hat{\textbf{m}} | \Psi _0 \rangle \right] \end{aligned}$$
  where $\hat{\mu }$ is the electric dipole operator, $\hat{\textbf{m}}$ is the magnetic dipole operator, $\hat{\textbf{p}}$ is the linear momentum operator, and $\Delta E_{0 \rightarrow f}$ is the excitation energy. The rotational strength is reported in cgs units (10$^{-40}$ erg-esu-cm/Gaussian), which are conventional for ECD spectroscopy. Gauge invariance is satisfied when $R^{\text {(len)}}$ and $R^{\text {(vel)}}$ are numerically equivalent in the complete basis set limit; discrepancies between the two representations provide an indication of basis set incompleteness or deficiencies in the electronic structure method.
- **Simulated spectra:** Continuous spectra were generated by convoluting the discrete set of transitions with Gaussian broadening functions:
  4 $$\begin{aligned} \varepsilon (\omega ) = \sum _{f} \frac{1}{\sqrt{2\pi \sigma ^2}} R_{0\rightarrow f} \exp \left[ -\frac{(\omega - \Delta E_{0\rightarrow f})^2}{2\sigma ^2}\right] \end{aligned}$$
  where $\varepsilon (\omega )$ is the ECD intensity at frequency $\omega$, $\Delta E_{0 \rightarrow f}$ is the excitation energy for transition *f*, and $\sigma$ is the standard deviation of the Gaussian broadening. The full width at half-maximum (FWHM) is related to $\sigma$ by $\text {FWHM} = 2\sqrt{2\ln 2}\,\sigma$. A typical FWHM of 50 cm$^{-1}$ was employed to match experimental broadening.

The computed ECD spectra were compared with experimental data from the literature [9] to evaluate the performance of each functional. To ensure the reliability of the subsequent ECD calculations, the root-mean-square deviation (RMSD) was employed to quantify the agreement between calculated and experimental excitation energies.

## Results and discussion

### Structural properties

#### RMSD analysis of the Fe(acac)$_3$ complex and its CCl$_4$ adduct

##### *Structural analysis of the Fe(acac)*$_3$*complex*

The calculation of the root-mean-square deviation (RMSD) constitutes a fundamental quantitative tool for comparing molecular structures. For the tris(acetylacetonate)Fe(III) complex, Fe(acac)$_3$, a comparative analysis was performed between its experimentally determined X-ray crystallographic structure (at 0.84 Å resolution) [28] and the geometry optimized via density functional theory (DFT). The optimized structures are shown in Fig. 1, which presents the two enantiomeric forms obtained under D$_3$ symmetry constraints: the left-handed ($\Lambda$) and right-handed ($\Delta$) isomers. This comparison reveals pertinent information regarding the quality of the theoretical model. The RMSD was calculated according to the mathematical expression:

5 $$\begin{aligned} \text {RMSD} = \sqrt{\frac{1}{N} \sum _{i=1}^{N} \Vert \vec {v}_i - \vec {w}_i\Vert ^2}, \end{aligned}$$

where *N* represents the total number of atoms considered, $\vec {v}_i$ are the atomic coordinates of the experimental structure after superposition (alignment) procedures, and $\vec {w}_i$ correspond to the coordinates of the calculated structure. For the isolated Fe(acac)$_3$ system (43 atoms), a total RMSD of 1.354 Å  was obtained. This discrepancy, exceeding the experimental resolution of 0.84 Å, was primarily attributed to the inherent uncertainty in hydrogen atom positions and methyl group orientations in the crystallographic data, where low atomic scattering factors limit precision. In contrast, the coordination core analysis (comprising the *Fe* ion and six oxygen atoms) yielded an RMSD of only 0.082 Å. This value is an order of magnitude smaller than the experimental resolution, demonstrating that the DFT method reproduces the octahedral geometry with exceptional fidelity. The fact that the core RMSD is significantly below the 0.84 Å  threshold confirms that the theoretical model predicts primary coordination interactions with precision exceeding the discriminatory power of the diffraction data, validating its reliability for describing the metal–ligand framework. The decomposition of RMSD by atomic subsets corroborates this interpretation: for the 22 heavy atoms (Fe, O, C), the RMSD is 0.314 Å, which remains well below the experimental resolution, indicating excellent agreement for the overall heavy-atom framework. For the complete structure including all hydrogens, the RMSD rises to 1.354 Å, exceeding the experimental resolution. This progression evidences that the primary source of discrepancy resides in the peripheral atoms with greater thermal and conformational freedom, particularly hydrogen atoms, rather than in the central structural core of the complex.

##### *Structural analysis of the Fe(acac)*$_3 \cdot$*CCl*$_4$*adduct*

The inclusion of a carbon tetrachloride molecule (CCl$_4$) to form a charge-transfer adduct introduces structural complexities, resulting in a total RMSD of 1.284 Å  for the 48-atom system. For the 24 heavy atoms (C, O, Fe, Cl), the RMSD is 1.042 Å, still exceeding the experimental resolution of 0.84 Å  by approximately 0.2 Å. This persistent discrepancy was primarily attributed to the relative positioning of the CCl$_4$ molecule with respect to the metal complex. Unlike the inner coordination core, which is governed by strong electrostatic and covalent forces and remains exceptionally well-described (RMSD = 0.082 Å), the adduct is stabilized by weak van der Waals interactions. These are highly sensitive to environmental effects. While the DFT simulation was performed in the gas phase, the experimental data reflect solid-state crystal packing. Consequently, the theoretical model does not fully replicate the lattice forces that dictate the specific orientations of the CCl$_4$ molecule within the crystal.

The reduction in RMSD from 1.284 Å to 1.042 Å  upon excluding hydrogens confirms that, while light atoms contribute to the statistical noise, the primary deviation originates from the spatial arrangement between the two molecular units. This result is considered reasonable for gas-phase calculations of supramolecular assemblies. It highlights that the DFT model accurately predicts the metal–ligand framework with precision exceeding the experimental resolution, while the uncertainties in CCl$_4$ positioning reflect the inherent challenges of modeling non-covalent interactions without accounting for explicit crystal packing. The density functional employed, although potentially including corrections for dispersive interactions (D3BJ), may not accurately capture the energetic balance of these weak interactions in the solid-state context.

To complement the structural validation (RMSD = 0.082 Å), it has been estimated the energetic stability of the Fe(acac)$_3\cdot$CCl$_4$ adduct. The interaction energy was calculated at the M06/def2-TZVPP level, including basis set superposition error (BSSE) correction using the counterpoise method. The BSSE-corrected binding energy ($\Delta E_{int,BSSE}$) is −6.16 kcal/mol, defined as follows:

6 $$\begin{aligned} \Delta E_{int,BSSE} = E_{complex(CP)} - \left[ E_{Fe(acac)_3(CP)} + E_{CCl_4(CP)} \right] \end{aligned}$$

The raw (uncorrected) complexation energy is −7.45 kcal/mol, with a BSSE of 0.00204 hartree ($\approx$1.28 kcal/mol). The counterpoise-corrected total energy of the complex is −4189.67649 hartree, and the sum of corrected fragment energies is −4189.66667 hartree. The final corrected binding energy of −6.16 kcal/mol confirms the stability of the adduct and is consistent with the expected magnitude for non-covalent interactions (e.g., halogen bonding or van der Waals contacts) in similar supramolecular systems. This energetic validation supports the reliability of the chosen theoretical level, complementing the structural RMSD analysis.

The Cartesian coordinates of the optimized molecular geometries for Fe(acac)$_3$ (under $D_3$ point group symmetry) and for the adduct Fe(acac)$_3\cdot$CCl$_4$ are provided in the Supplementary Information, Tables S1 and S2, respectively. All geometries were optimized using the M06 functional, the def2-SVP basis set, and the DKH2 relativistic correction.

**Table 2:** Hirshfeld charges and spin densities (non-hydrogen atoms)

Index	Atom	Fragment	Role	Charge (*e*)	Spin density
*Iron center*					
14	Fe	—	Metal	0.394 288	3.975 079
*Coordinating oxygens*					
12	O	acac-1	enolate O	−0.203 893	0.136 882
13	O	acac-1	enolate O	−0.204 306	0.133 971
39	O	acac-2	enolate O	−0.208 146	0.120 081
40	O	acac-2	enolate O	−0.206 607	0.136 864
41	O	acac-2	enolate O	−0.199 093	0.123 525
42	O	acac-2	enolate O	−0.209 497	0.122 688
*Acac carbon framework (representative atoms)*					
5	C	acac-1	$\beta$-C	−0.101 356	0.032 185
25	C	acac-2	$\beta$-C	−0.101 287	0.029 365
26	C	acac-2	$\beta$-C	−0.102 538	0.032 075
4	C	acac-1	carbonyl-adj. C	0.132 881	0.011 138
0	C	acac-1	methyl C	−0.078 597	0.008 307
*CCl*$_4$ *molecule*					
43	C	CCl$_4$	central C	0.146 240	0.000 919
44	Cl	CCl$_4$	Cl	−0.041 169	0.000 517
45	Cl	CCl$_4$	Cl (proximal)	−0.002 187	0.001 932
46	Cl	CCl$_4$	Cl	−0.041 114	0.000 450
47	Cl	CCl$_4$	Cl	−0.052 536	0.000 084
Total				−0.000 080	4.999 996

**Fig. 2:**
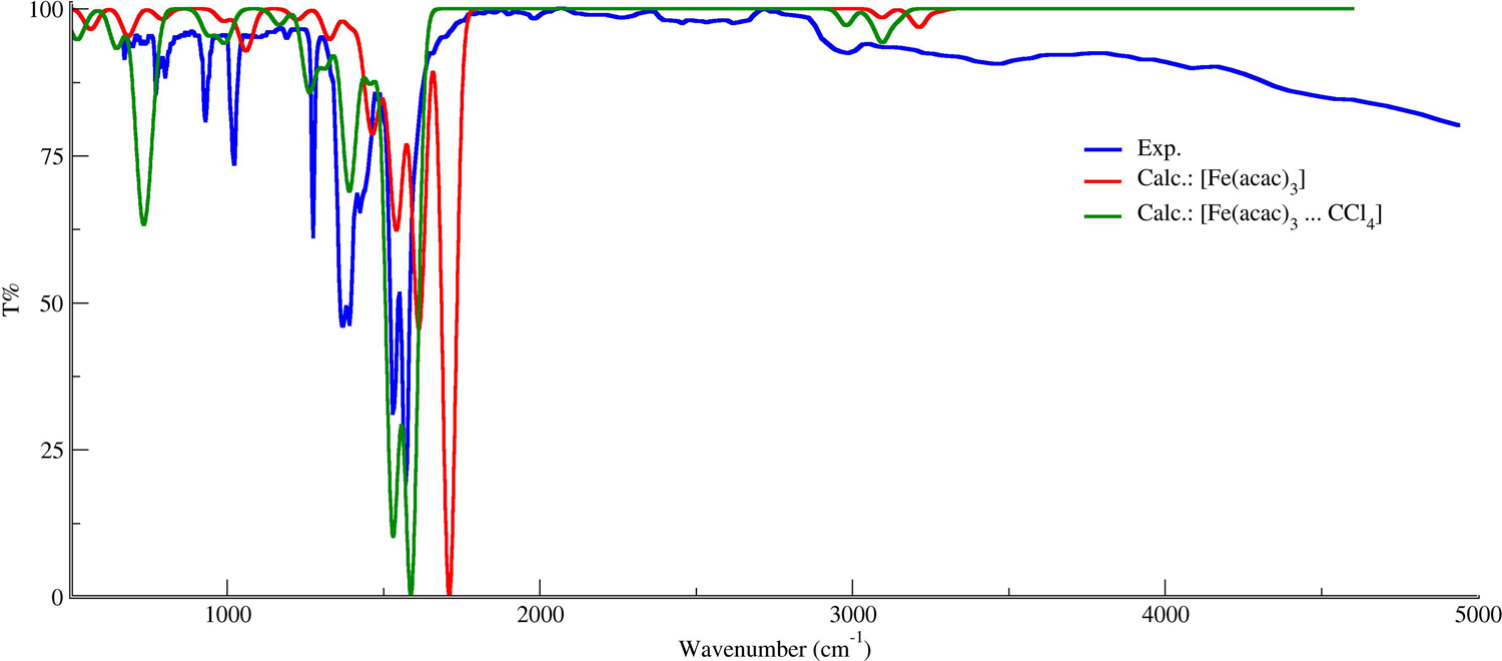
Experimental IR spectrum of Fe(acac)$_3$ (blue line) compared to the M06/def2-SVP + DKH2 calculated spectra of the parent complex (red line) and the CCl$_4$ adduct (dark green line)

#### Hirshfeld population analysis of the Fe(acac)$_3 \cdot$CCl$_4$ complex

Table 2 presents the Hirshfeld partial charges and spin densities for all non-hydrogen atoms. The total integrated spin of $\approx 5.000\,e$ is unambiguously consistent with a high-spin $d^5$ Fe iii center ($S = 5/2$).

##### Iron center and spin distribution

The Fe atom carries a Hirshfeld partial charge of $+0.394\,e$, well below the formal $+3$ value—a known feature of Hirshfeld partitioning, which distributes charge more conservatively than NPA or Mulliken analysis. The spin density on Fe (3.975, $\approx 79.5\%$ of the total) confirms the high-spin $d^5$ configuration. The remaining spin is largely located on the six coordinating oxygens ($\rho _s = 0.120$–0.137 each; $\approx 14.8\%$ collectively), evidencing a covalent component in the Fe–O bond through partial delocalisation of unpaired electron density into the enolate $\pi$-system.

##### Acac ligand

The carbon skeleton displays a clear alternating charge pattern dictated by the enolate electronic structure: carbonyl-adjacent $\alpha$-carbons are positively charged ($\approx +0.132\,e$), while the central $\beta$-carbons and methyl groups are negatively charged ($\approx -0.101$ and $-0.078\,e$, respectively). The $\beta$-carbons stand out by exhibiting the highest spin densities among all ligand carbons ($\rho _s \approx 0.030$–0.032), consistent with spin delocalisation through the conjugated six-membered chelate ring.

##### CCl$_4$ adduct molecule

The CCl$_4$ molecule is magnetically innocent: all spin densities are essentially zero ($\rho _s < 0.002$), with no measurable spin transfer from the Fe iii center. The central carbon is strongly positive ($+0.146\,e$), reflecting the electron-withdrawing effect of the four chlorines. Notably, one chlorine (Cl$_{45}$, $-0.002\,e$) is nearly neutral compared to the other three ($-0.041$ to $-0.053\,e$). This asymmetry indicates a directional polarization: the proximal Cl is oriented toward the Fe center, and the resulting electrostatic interaction reduces the charge transfer from C to that particular Cl.

The total spin density sums to $\approx$ 5.000 with the Fe center accounting for $\approx$79.5% and the six coordinating oxygens together contributing $\approx$14.8%, leaving a smaller residual on the carbon framework. This is consistent with a predominantly ionic but partially covalent Fe–O bonding picture in a high-spin ferric tris-chelate complex.

#### Infrared spectra of Fe(acac)$_3$ and the Fe(acac)$_3 \cdot$CCl$_4$ adduct

The accompanying Fig. 2 compares the experimental IR spectrum of the isolated Fe(acac)$_3$ complex (blue line) with the M06/def2-SVP + DKH2 calculated spectra of the parent complex (red line) and the CCl$_4$ adduct, Fe(acac)$_3\cdot$CCl$_4$ (dark green line). This combined experimental and theoretical approach helps elucidate the nature of the interaction between the complex and the CCl$_4$ molecule.

The experimental spectrum, acquired from a KBr pellet and sourced from the NIST database [29], is displayed in blue. This serves as the reference for the vibrational signature of the pristine complex. The calculated spectra, obtained at the M06/def2-SVP level of theory including the DKH2 relativistic correction, are shown in red for the isolated Fe(acac)$_3$ complex (in its optimized $D_3$ symmetry) and in green for the Fe(acac)$_3 \cdot$CCl$_4$ adduct. This computational method provides a reliable basis for assigning spectral features and interpreting the electronic and structural effects of adduct formation.

A preliminary visual inspection of the spectra across the entire mid-infrared range (400–5000 cm$^{-1}$) reveals a high degree of qualitative agreement between the experimental and calculated spectra for the isolated complex. The positions and relative intensities of the major vibrational bands are well-reproduced by the theoretical model, validating the chosen computational approach. The calculated spectrum for the adduct, while broadly similar, exhibits distinct shifts in key vibrational modes, indicative of a specific interaction between Fe(acac)$_3$ and CCl$_4$.

The most informative spectral region for probing this interaction lies in the higher wavenumber range, specifically around 1600 cm$^{-1}$. As highlighted in point (1), the vibrational transition calculated at 1641 cm$^{-1}$ for the isolated Fe(acac)$_3$ complex corresponds to a normal mode primarily associated with the acac ligands, likely a mixed C=O and C=C stretching vibration characteristic of the $\beta$-diketonate ring. Upon adduct formation, this mode undergoes a significant redshift, appearing at 1588 cm$^{-1}$ in the calculated spectrum of Fe(acac)$_3 \cdot$CCl$_4$.

This 53 cm$^{-1}$ shift to a lower wavenumber suggests a weakening of the bonds involved in this vibration. This can be rationalized by the donation of electron density from the electron-rich *acac* ligands toward the CCl$_4$ molecule: this charge transfer slightly populates the antibonding orbitals of the ligand framework, consequently reducing the bond order and force constants. Thus, CCl$_4$ acts as a weak Lewis acid in this adduct.

Further evidence for adduct formation is provided by the vibrational modes of the CCl$_4$ moiety itself. As noted in point (2), the mode associated with the carbon-chlorine stretching vibration in the adduct is calculated at 728.8 cm$^{-1}$. The appearance and precise position of this band serve as a direct probe of the perturbation experienced by the CCl$_4$ molecule. The presence of this distinct feature in the adduct’s calculated spectrum, absent in that of the isolated complex, confirms the formation of a supramolecular entity. The specific frequency of this mode reflects the altered electronic environment and geometry of CCl$_4$ when interacting with the metal complex.

**Fig. 3:**
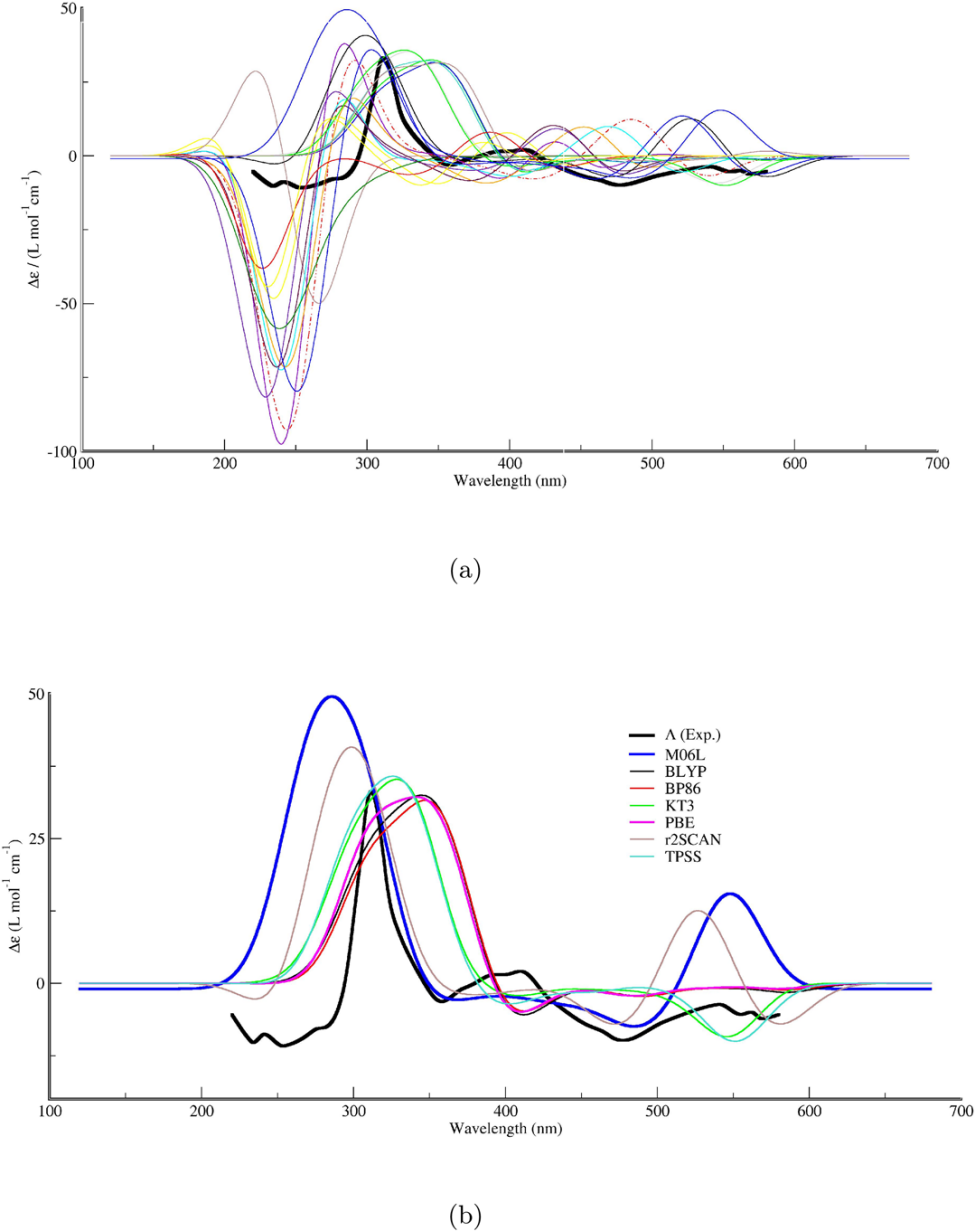
Benchmark of TD-DFT/def2-TZVPP for the ECD spectrum of $\Lambda$-Fe(acac)$_3\cdot$CCl$_4$. **a** Calculated spectra for 20 exchange-correlation functionals versus experiment (bold line). **b** Detailed view of the functionals yielding the closest agreement with the experimental curve

In summary, the combined experimental and theoretical spectral analysis confirms the formation of a distinct Fe(acac)$_3 \cdot$CCl$_4$ adduct. The characteristic shift of the ligand-based vibration from 1641 to 1588 cm$^{-1}$ points to an electronic interaction where CCl$_4$ withdraws electron density from the acac ligands. This interaction is further corroborated by the appearance of a perturbed C–Cl stretching mode for the bound CCl$_4$ at 728.8 cm$^{-1}$. The excellent agreement between the experimental and calculated spectra for the isolated complex lends strong support to the theoretical assignment of these interaction-induced spectral changes.

### ECD spectra of the $\Lambda$-Fe(acac)$_3\cdot$CCl$_4$ adduct

Figure 3a presents the electronic circular dichroism (ECD) spectra of the adduct formed between the $\Lambda$-enantiomer of Fe(acac)$_3$ and CCl$_4$. The experimental spectrum, shown in black [9], serves as the benchmark for evaluating the performance of various theoretical approaches. Superimposed on the experimental trace are computed ECD spectra obtained from time-dependent density functional theory calculations employing 20 different functionals. These are representative of the major functional families, including hybrid, meta-hybrid, range-separated, and pure generalized gradient approximation functionals.

The experimental spectrum displays a distinct negative Cotton effect across the entire wavelength range (approximately 200–600 nm), which is consistent with the expected chiroptical signature for the $\Lambda$-configuration of this chiral metal complex. The molar circular dichroism ($\Delta \varepsilon$) values become increasingly negative starting from the onset around 600 nm, reaching approximately $-100 \times 10^{-4}$ L$\cdot$mol$^{-1}\cdot$cm$^{-1}$ at the lower wavelength limit of 200 nm. This relatively featureless, monotonically decreasing profile is characteristic of the electronic transitions in this system and its interaction with the CCl$_4$ molecule.

A comparison between the experimental data and the TD-DFT calculated curves reveals considerable variation in the predictive capacity of the selected functionals. Several key observations can be made. First, regarding qualitative agreement, most of the tested functionals successfully reproduce the overall negative sign of the Cotton effect observed experimentally for the $\Lambda$-isomer. This indicates that TD-DFT, regardless of the specific functional, can capture the inherent chiral nature of the electronic transitions in the $\Lambda$-Fe(acac)$_3\cdot$CCl$_4$ adduct.

Second, despite the qualitative agreement in sign, substantial quantitative differences exist among the functionals and between the calculations and the experiment. Concerning the magnitude of $\Delta \varepsilon$, while many calculated curves align reasonably well with the experimental order of magnitude ($10^{-4}$ L$\cdot$mol$^{-1}\cdot$cm$^{-1}$), one particular functional overestimates the ECD intensity for the $\Lambda$-isomer by approximately tenfold. This highlights a critical sensitivity of the calculated ECD intensity to the choice of functional or computational parameters, particularly when modeling adduct formation.

Regarding spectral shape and onset, many functionals yield nearly identical, linearly decreasing $\Delta \varepsilon$ values. However, the experimental spectrum’s shape, while generally linear, possesses subtle features and a specific slope that certain functionals reproduce more accurately than others. A more detailed, wavelength-resolved analysis would be necessary to assess the agreement in spectral features, such as shoulders or peak positions, that might arise from the adduct interaction and are characteristic of the $\Lambda$-enantiomer.

The benchmark table reveals a striking dichotomy among DFT functionals for ECD calculations in the 200–400 nm range. This spectral window was specifically chosen because it encompasses the highest experimental ECD intensity values. On one hand, several functionals—including PBE, BP86, BLYP, TPSS, r2SCAN, B3LYP, PBE0, M06, and M06L—successfully reproduce the experimental profile, which is characterized by an intense negative Cotton effect at short wavelengths, a sigmoidal zero-crossing, and a positive band at longer wavelengths. Among these, the PBE functional proves to be one of the most reliable and balanced choices for ECD calculations in this range, excelling precisely where many more sophisticated functionals fail. A more comprehensive analysis, including all quantitative metrics and detailed comparisons, is provided in the Supplementary Information (Table S3).

### Comparative analysis of density functionals and relativistic effects

Figure 3b presents the ECD spectra of the adduct formed between the $\Lambda$-enantiomer of Fe(acac)$_3$ and CCl$_4$, highlighting the performance of selected functionals in comparison to the experimental results. The experimental spectrum, depicted in black, serves as the reference for evaluating the theoretical calculations. Among the various functionals tested—including M06-L, BLYP, BP86, KT3, PBE, r2SCAN, and TPSS—the PBE functional demonstrates the most satisfactory agreement with the experimental data across the entire analyzed wavelength range.

**Fig. 4:**
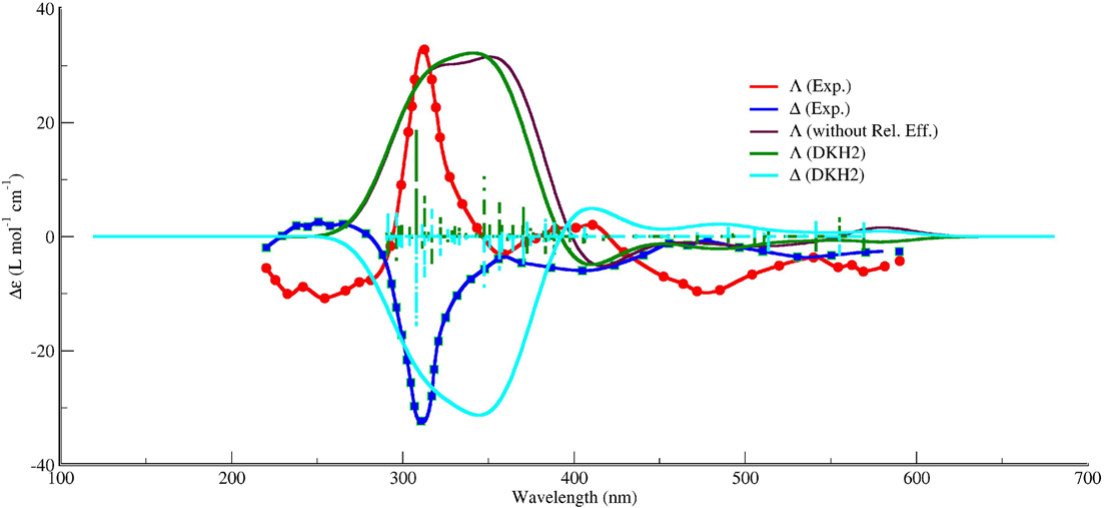
Experimental and calculated ECD spectra of $\Delta$- and $\Lambda$-Fe(acac)$_3\cdot$CCl$_4$. The experimental spectra were digitized [30] from Ref. [9] and are compared with spectra calculated at the TD-PBE(D3BJ)/def2-TZVPP level. The calculated spectra for both enantiomers include relativistic effects via the DKH2 Hamiltonian. For comparison, the calculated spectrum for the $\Lambda$ isomer without relativistic corrections (i.e., excluding DKH2) is also shown

Upon examining the calculated spectra, it becomes evident that the PBE functional reproduces the experimental curve with remarkable fidelity. Not only does it correctly capture the overall sign of the Cotton effect, but it also accurately predicts the magnitude and position of the main spectral features. The characteristic negative band centered near 350 nm is well-reproduced by PBE, as are the subsequent variations in the 400–600 nm region. In contrast, other functionals show significant deviations. The M06-L functional, for instance, while capturing some features, exhibits discrepancies in intensity at key wavelengths. The BLYP and BP86 functionals fail to reproduce the complex profile, showing essentially flat responses that do not correspond to experimental observations. Similarly, KT3, r2SCAN, and TPSS demonstrate limited success, with TPSS showing some activity only at longer wavelengths but failing to capture essential features in the higher-energy region.

The superior performance of the PBE functional can be attributed to several inherent characteristics that make it particularly well-suited for studying excited states in transition metal complexes. As a generalized gradient approximation functional, PBE incorporates both the electron density and its gradient, providing a more accurate description of electronic distributions compared to simpler local density approximations. This is crucial for transition metal complexes like Fe(acac)$_3$, where *d*-orbitals participate in electronic transitions and require a balanced treatment of exchange and correlation effects.

Furthermore, the PBE functional offers a favorable balance between computational efficiency and accuracy, making it a practical choice for relatively large systems involving organic ligands and explicit solvent interactions. Its performance for excited states, particularly regarding chiroptical properties, has been validated across numerous studies, establishing it as a reliable tool for predicting and interpreting ECD spectra.

Figure 4 presents the ECD spectra for the $\Lambda$- and $\Delta$-stereoisomers of the Fe(acac)$_3\cdot$CCl$_4$ adduct, comparing experimental solid-state measurements with gas-phase PBE calculations, both with and without scalar relativistic effects via the DKH2 Hamiltonian. The experimental spectra exhibit the characteristic mirror-image relationship expected for enantiomers, with the $\Lambda$-isomer displaying a predominantly negative Cotton effect and the $\Delta$-isomer showing the corresponding positive features.

The PBE calculations without relativistic corrections capture the overall sign pattern but show discrepancies in band intensities and positions. The inclusion of DKH2 scalar relativistic effects noticeably improves the agreement with experiment, particularly in reproducing intensity ratios and band shapes. However, residual discrepancies remain due to the differing physical states: experimental data were acquired in the solid state, where intermolecular interactions and crystal field effects influence the electronic structure, whereas calculations were performed for isolated gas-phase molecules.

### Electronic transitions and orbital assignments

**Fig. 5:**
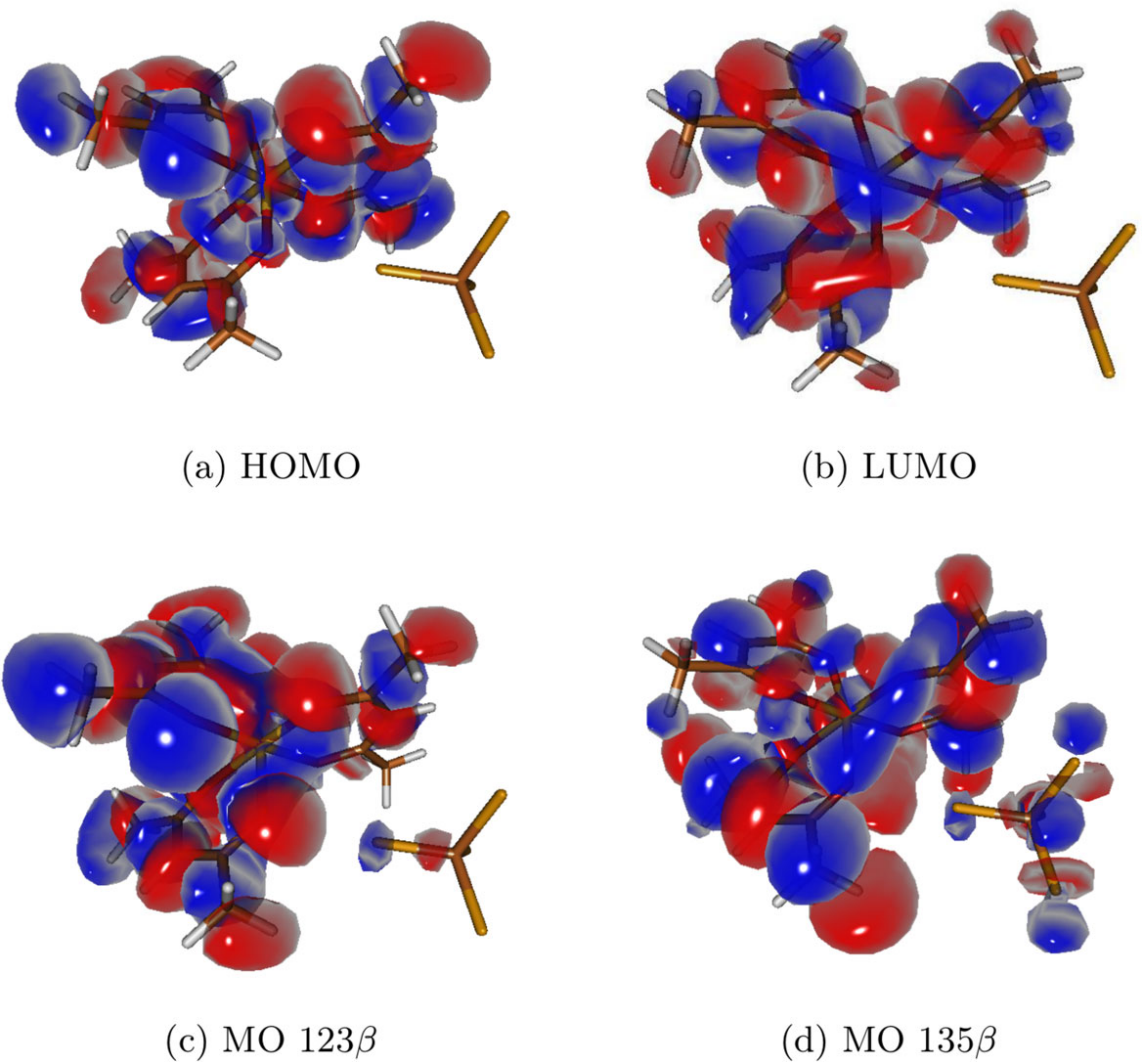
Frontier molecular orbitals and key transition orbitals for $\Lambda$-Fe(acac)$_3\cdot$CCl$_4$. (**a**) HOMO, (**b**) LUMO, and (**c**, **d**) the dominant orbital pair contributing to the most intense Cotton effect in the calculated ECD spectrum

Intense circular dichroism (CD) signals are observed in the UV region, corresponding to ligand-centered ($\pi \rightarrow \pi ^*$) transitions and charge-transfer (CT) bands. The main spectral features have been assigned to specific electronic transitions. For the $\Lambda$-Fe(acac)$_3\cdot$CCl$_4$ adduct, the band at approximately 570 nm corresponds to the HOMO–LUMO transitions (Fig. 5a, b), whereas the more intense band is centered around 370 nm is associated with the dominant orbital pair contributing to the Cotton effect—from orbital 123$\beta$ to orbital 135$\beta$—as illustrated in Fig. 5c, d.

The 570 nm band exhibits greater sensitivity to relativistic effects due to the significant involvement of metal *d*-orbitals in the frontier molecular orbitals (FMOs). In the case of the transition associated with the 370 nm band, there is strong participation from the CCl$_4$ moiety. This indicates that the transition involves not only the orbitals of the metal complex but also significant interactions with the carbon tetrachloride molecule, which acts as either a solvent or an external ligand. This contribution from CCl$_4$ influences both the intensity and the electronic nature of the transition observed in this spectral region.

**Fig. 6:**
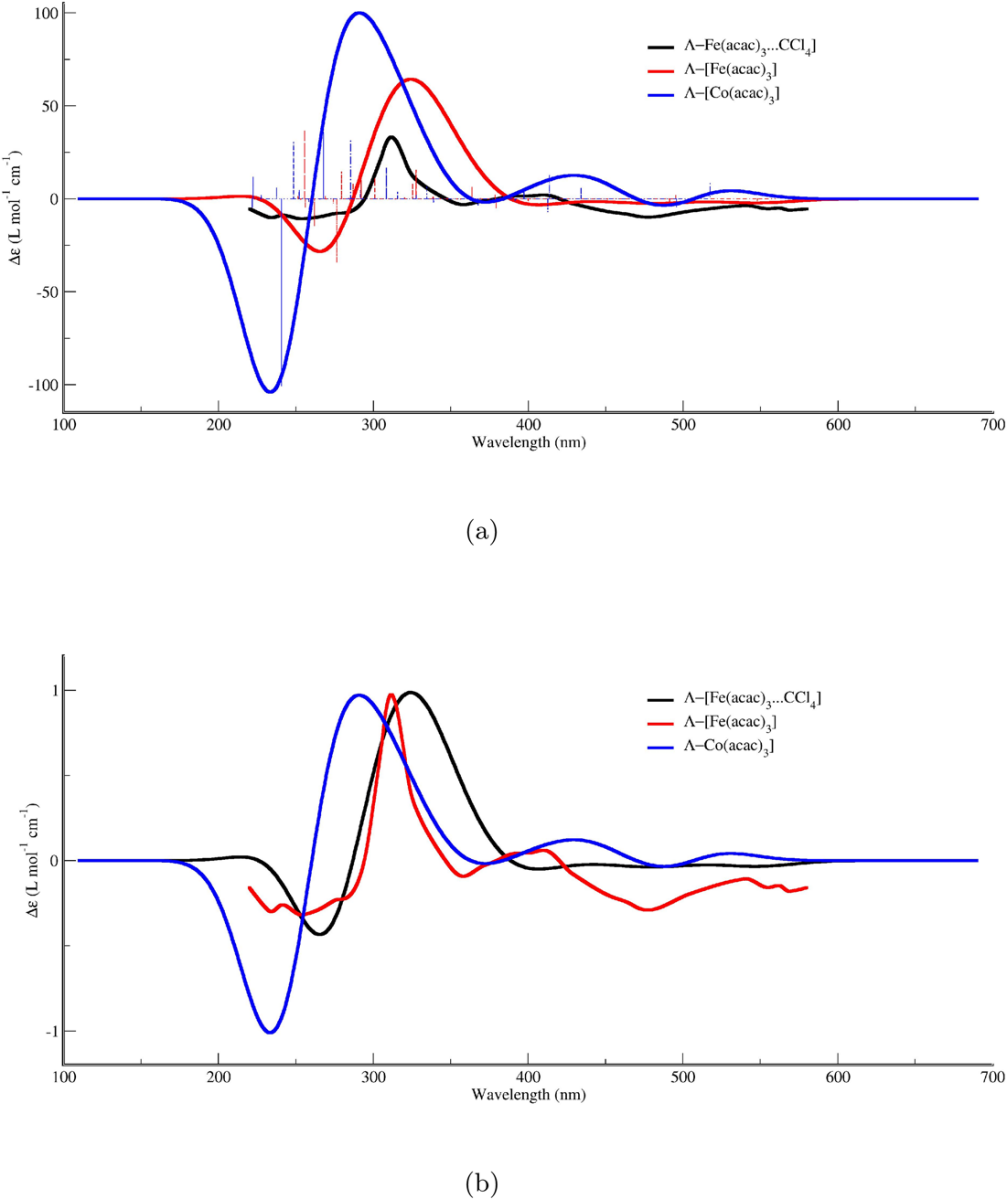
(**a**) Experimental ECD spectrum of the $\Lambda$-Fe(acac)$_3\cdot$CCl$_4$ adduct (black line) compared to spectra calculated at the TD-PBE-(D3BJ)/def2-TZVPP + DKH2 level for the bare $D_3$-symmetric Fe(acac)$_3$ and Co(acac)$_3$ complexes. (**b**) The same set of spectra after intensity normalization

### Natural Transition Orbital (NTO) analysis for Fe(acac)$_3\cdot$CCl$_4$

To rationalize the nature of the electronic excitations, Natural Transition Orbital (NTO) analysis was carried out for the first 20 excited states of the Fe(acac)$_3\cdot$CCl$_4$ adduct at the PBE/def2-TZVPP level including D3(BJ) dispersion and DKH2 relativistic corrections. For each state, the NTO approach diagonalizes the transition density matrix, providing the most compact orbital representation of the electron hole and particle. Table S4 lists the excitation energies, the dominant NTO occupation numbers, and the leading single-excitation contributions extracted from the TD-DFT/TDA output. States 1–14 are characterized predominantly by $\beta$-spin excitations (mainly 126b $\rightarrow$ 127b), indicative of metal-centered d-d transitions. In contrast, from State 15 onward, $\alpha$-spin excitations (e.g., 131a $\rightarrow$ 132a) become dominant, signaling a change in the transition character. The NTO occupation numbers are close to unity for the primary pair in most cases, confirming that these excited states are well described by a single configuration.

### Theoretical analysis of the ECD for Fe and Co complexes

The analysis of the electronic circular dichroism (ECD) spectra presented in Fig. 6a, b provides insight into the electronic transitions and chiral interactions of the metal complexes $\Lambda$-Fe(acac)$_3$ and $\Lambda$-[Co(acac)$_3$], calculated using the TD-PBE method. The spectra are depicted in two formats: one displaying the raw differential absorption and the other presenting normalized curves for a clearer comparative assessment.

$\Lambda$-Fe(acac)$_3$ complex (red line) exhibits distinct ECD bands, with a significant positive Cotton effect observed around 300–350 nm and a negative band near 250–275 nm. The $\Lambda$-[Co(acac)$_3$] complex (blue line) shows a similar spectral profile but with notable differences in intensity; the positive band for the cobalt complex is more pronounced, particularly in the 300–350 nm region, and its negative band near 275 nm is substantially more intense. This suggests a stronger chiral perturbation or different electronic configurations arising from the metal center.

The most striking feature is the black curve, which represents the ECD spectrum of the $\Lambda$-Fe(acac)$_3 \cdot$CCl$_4$ adduct. Upon interaction with carbon tetrachloride, the ECD signal of the iron complex undergoes significant modifications. In the longer-wavelength region (around 350–500 nm), a new, broad, and relatively weak positive feature emerges in the adduct, which is nearly silent for the isolated complexes. Conversely, in the high-energy region (around 275 nm), the negative band of the adduct is substantially attenuated compared to the isolated $\Lambda$-Fe(acac)$_3$.

This spectral perturbation indicates that the non-covalent interaction with CCl$_4$ induces a change in the electronic environment and the chiral disposition of the acac ligands around the iron center. The normalization of the spectra confirms that these are not mere scaling effects but rather genuine alterations of the rotatory strengths of specific electronic transitions. The data collectively suggest that the CCl$_4$ molecule selectively influences the electronic transitions of the metal complex, likely through weak intermolecular interactions that modify the symmetry and the magnetic dipole–electric dipole coupling terms responsible for the observed Cotton effects.

## Conclusions

The experimental ECD spectrum of the $\Lambda$-Fe(acac)$_3\cdot$CCl$_4$ adduct constitutes a rigorous benchmark for TD-DFT methodologies. This comparative analysis reveals that, although most functionals correctly predict the sign of the Cotton effect for this enantiomer, the accuracy in reproducing the spectral lineshapes and intensities varies considerably. Such discrepancies, underscored by the marked overestimation of intensity by specific functionals, highlights the critical importance of benchmarking theoretical methods against experimental data for transition metal complexes and their adducts to ensure a reliable interpretation of chiroptical properties.

Among the tested functionals, PBE—when employed with DKH2 scalar relativistic corrections—emerges as the most suitable choice among those evaluated within the present computational framework. Its capability to faithfully reproduce not only the sign but also the intricate spectral features of the experimental spectrum substantiates its robustness for excited-state studies of transition metal complexes. The reliability of the PBE functional is further corroborated by its performance in analogous systems. While TD-PBE calculations qualitatively describe the ECD spectral profile for the $\Lambda$-[Co(acac)$_3$] complex and reproduce its characteristic shape, notable discrepancies in band intensities are observed, with the positive band being significantly more pronounced for the cobalt complex. The success of the PBE functional is therefore attributable to its balanced theoretical foundation and non-empirical construction, demonstrating that judicious functional selection, within the limits of the methods tested here, is critical for reliable computational chiroptical spectroscopy.

While the inclusion of relativistic effects proves essential for the accurate modeling of this iron complex, the remaining discrepancies between calculated and experimental spectra suggest that environmental factors, such as solid-state versus gas-phase effects, impose inherent limitations on achieving full quantitative agreement. Furthermore, while the optimized geometry shows excellent agreement with the experimental crystal structure (RMSD = 0.082 Å), a systematic analysis of ECD sensitivity to geometric variations was beyond the scope of this work and will be addressed in a future study.

**Supplementary Information** SI accompanies this paper and is available in a separate PDF file.

## Supplementary Information

Below is the link to the electronic supplementary material.

Supplementary file 1 (pdf 364 KB)

## Data Availability

The data that support the findings of this study are available from the corresponding author upon reasonable request.
