# Supplementary material for: Assessment of DFT functionals for chiroptical properties of Fe(acac)3 adduct: a study of M(acac)3 (M = Fe and Co) stereoisomers
Source: J Mol Model. 2026 May 18;32(6):192. doi: 10.1007/s00894-026-06767-8 (PMC13180779; doi:10.1007/s00894-026-06767-8)
Supplement: Supplementary file 1 — (pdf 364 KB) [file 894_2026_6767_MOESM1_ESM.pdf]

Assessment of DFT Functionals for Chiroptical Properties of $Fe(acac)_3$ Adduct: A Study of $M(acac)_3$ (M = Fe and Co) Stereoisomers

[1,*]Nelson H. Morgon

[1,*]Institute of Chemistry, UNICAMP, Cidade Universitária - "Prof. Zeferino Vaz", Campinas, 13.083-861, São Paulo, Brazil.

Contributing authors: nhmorgon@unicamp.br;

Supplementary Information

Table S1. Symmetry-unique Cartesian coordinates (in Å) for Λ-$Fe(acac)_3$ under D_3 point group symmetry, obtained at the M06/def2-SVP + DKH2 level of theory.

Atom	x	y	z
Fe	0.00000	0.00000	0.00000
O	1.45683	0.77219	1.15332
C	2.71305	0.68657	1.03001
C	3.37746	0.00000	0.00000
C	3.51293	1.38607	2.08651
H	3.22138	2.44840	2.11082
H	3.24667	0.96665	3.07027
H	4.47048	0.00000	0.00000
H	4.59793	1.30436	1.93367

Table S2. Cartesian Coordinates (in Å) for the Λ-Fe(acac)$_3$·CCl$_4$ adduct obtained at M06/def2-SVP + DKH2 level of theory.

Atom	x	y	z
Fe	8.80074	-4.75251	5.21188
Cl	5.80965	1.01538	3.26486
Cl	7.11624	-1.49881	3.99637
Cl	7.49442	0.88661	5.64816
Cl	8.70656	0.74677	2.99554
O	10.36211	-5.70282	6.06215
O	7.05876	-4.19172	4.33153
O	7.61988	-5.94222	6.39466
O	8.84152	-6.24912	3.80557
O	8.86768	-3.28493	6.57297
O	9.89306	-3.55191	4.02648
C	10.03662	-7.85890	5.09826
C	10.32403	-1.73270	5.50319
C	10.45391	-2.43108	4.28006
C	10.59111	-6.96127	6.04011
C	11.27480	-1.84321	3.15642
C	11.53150	-7.47231	7.10791
C	4.99972	-4.38998	3.18160
C	5.69489	-6.02833	4.98784
C	5.98707	-4.88434	4.21254
C	6.05873	-7.64061	6.91137
C	6.50655	-6.47044	6.06343
C	7.27702	0.27190	3.97473
C	8.76326	-8.46761	2.99147
C	9.22504	-7.44932	4.01086
C	9.39731	-1.35613	7.83192
C	9.52729	-2.18813	6.57798
H	10.00531	-0.43373	7.80121
H	10.31692	-8.91871	5.16593
H	10.60193	-1.63540	2.29939
H	10.83602	-0.76766	5.60774
H	11.06170	-7.31487	8.10114
H	11.77960	-8.54290	6.98805
H	11.79040	-0.90932	3.44547
H	12.01631	-2.59211	2.81246
H	12.46182	-6.86941	7.09840
H	4.08798	-5.01231	3.12559
H	4.71959	-3.34231	3.41686

H	4.74501	-6.54842	4.80547
H	5.06299	-8.02751	6.62697
H	5.49551	-4.36812	2.18940
H	6.04984	-7.33722	7.97782
H	6.81046	-8.45183	6.81855
H	7.65366	-8.4727	2.97265
H	8.33074	-1.08500	7.97066
H	9.09531	-8.15110	1.98167
H	9.13418	-9.48833	3.19870
H	9.69078	-1.96870	8.70858

General Comments on the Benchmark Data

The benchmark table presented below reveals a striking dichotomy among DFT functionals for ECD calculations in the 200–400 nm range (this spectral window was specifically chosen because it encompasses the highest experimental ECD intensity values). On one hand, several functionals–including PBE, BP86, BLYP, TPSS, r2SCAN, B3LYP, PBE0, M06, and M06L–successfully reproduce the experimental profile characterized by an intense negative Cotton effect at short wavelengths, a sigmoidal zero-crossing, and a positive band at longer wavelengths. These functionals also exhibit perfect sign agreement for all Cotton effects, with MAE values ranging from 13.8 nm (BP86) to 21.2 nm (PBE0). On the other hand, a subset of functionals–notably DSD-PBEB95, KT3, CAM-B3LYP, and LC-BLYP–fail catastrophically in qualitative terms. Despite showing deceptively good numerical MAE values (as low as 11.9 nm for DSD-PBEB95), these functionals produce entirely positive spectra, unphysical oscillations, or severely distorted profiles that do not resemble the experimental data at all. This underscores a critical lesson: numerical metrics alone are insufficient; profile fidelity and sign agreement are paramount in ECD spectroscopy.

The PBE functional demonstrates itself as one of the most reliable and balanced choices for ECD calculations in the 200–400 nm range, excelling precisely where many sophisticated functionals fail. While DSD-PBEB95, KT3, and CAM-B3LYP produce qualitatively incorrect spectra – showing entirely positive bands, unphysical oscillations, or distorted profiles – PBE faithfully reproduces the experimental signature: an intense negative Cotton effect at 200–250 nm, a clean sigmoidal zero-crossing, a positive band at 300–350 nm, and a gradual decay to baseline. With a mean absolute error of 14.6 nm, perfect sign agreement for all Cotton effects, and low computational cost (GGA level), PBE offers an ideal balance of accuracy and efficiency. More importantly, unlike double-hybrids or long-range corrected functionals that risk catastrophic sign inversions, PBE avoids artificial artifacts entirely. For routine configurational assignment of chiral molecules via ECD, PBE is therefore a robust, trustworthy, and cost-effective default choice.

Among the functionals that correctly reproduce the profile, BP86 achieves the lowest MAE (13.8 nm), followed closely by BLYP (14.0 nm), B3LYP (14.4 nm), and PBE (14.6 nm). The meta-GGA functionals (TPSS, r2SCAN, M06L) and hybrids (PBE0, M06) show slightly higher MAE values (15.9–21.2 nm) but remain acceptable for qualitative work. SCAN0, ωB97X, MN15, and MN15L show partial agreement, with MN15L exhibiting the highest MAE among profile-correct functionals (28.6 nm) and only partial sign agreement. In summary, for routine ECD applications, GGAs like PBE and BP86 offer an excellent compromise between accuracy, robustness, and computational cost, outperforming more sophisticated functionals that fail to capture the essential physics of the system.

Table S3. Complete Benchmark of ECD Data (200–400 nm): Experimental *vs.* All DFT Functionals.

Functional	MAE (nm)	Max Error (nm)	Sign Agreement	MAE Intensity	Follows Exp. Profile?
**Functionals That Correctly Reproduce the Experimental Profile**					
PBE	14.6	29.4	Yes	1.51	Yes
BP86	13.8	27.9	Yes	1.43	Yes
BLYP	14.0	28.3	Yes	1.46	Yes
TPSS	15.9	32.1	Yes	1.68	Yes
r2SCAN	16.4	33.0	Yes	1.72	Yes
B3LYP	14.4	29.0	Yes	1.49	Yes
PBE0	21.2	42.5	Yes	2.24	Yes
M06	17.8	35.7	Yes	1.88	Yes
M06L	19.3	38.0	Yes	2.05	Yes
TPSSH	18.7	36.5	Yes	1.95	Yes
SCAN0	24.1	48.3	Yes	2.78	Partial
ωB97X	22.3	44.2	Yes	2.41	Partial
MN15	25.4	50.1	Yes	2.96	Partial
MN15L	28.6	55.2	Partial	3.45	No
**Functionals That FAIL to Reproduce the Experimental Profile**					
DSD-PBEB95	11.9	24.0	No	1.02	No (positive only)
KT3	12.5	25.8	No	1.09	No (positive only)
CAM-B3LYP	15.2	30.6	No	1.58	No (oscillatory)
LC-BLYP	32.1	61.5	Partial	3.89	No (distorted)

Note: MAE = Mean Absolute Error. Sign Agreement indicates whether the sign of Cotton effects matches experiment. Intensity MAE in arbitrary units (same scale as experimental data).

Table S4. Excitation energies, dominant NTO occupations, and principal single-excitation contributions (weight > 0.1) for the first 20 excited states of the Λ-$Fe(acac)_3 \cdot CCl_4$ adduct at the PBE-D3(BJ)/def2-TZVPP level including DKH2 relativistic corrections.

State	Energy (eV)	Energy (cm^{-1})	Dominant NTO hole → particle (occupation)	Main single-excitation contributions (weight)
1	1.595	12863	126b → 127b (0.994)	126b → 127b (0.972)
2	1.678	13534	126b → 127b (0.988)	125b → 127b (0.970)
3	1.734	13984	126b → 127b (0.981)	124b → 127b (0.950)
4	1.807	14573	126b → 127b (0.967)	126b → 128b (0.838)
5	1.853	14945	126b → 127b (0.781)	126b → 129b (0.445), 125b → 129b (0.209), 123b → 127b (0.146), 125b → 128b (0.144)
6	1.877	15137	126b → 127b (0.818)	123b → 127b (0.812)
7	1.940	15646	126b → 127b (0.655)	124b → 128b (0.540)
8	1.946	15695	126b → 127b (0.650)	125b → 129b (0.263), 124b → 128b (0.228), 126b → 129b (0.210), 125b → 128b (0.157)
9	2.019	16287	126b → 127b (0.696)	125b → 128b (0.354), 124b → 129b (0.230), 125b → 129b (0.194)
10	2.067	16671	126b → 127b (0.961)	123b → 128b (0.760)
11	2.099	16926	126b → 127b (0.978)	123b → 129b (0.846)
12	2.177	17562	Mixed: 126b → 127b (0.456), 125b → 128b (0.286), 124b → 129b (0.222)	124b → 129b (0.335), 122b → 127b (0.204), 125b → 128b (0.110)
13	2.233	18010	126b → 127b (0.807)	122b → 127b (0.757)
14	2.289	18466	126b → 127b (0.965)	121b → 127b (0.958)
15	2.319	18705	131a → 132a (0.993)	131a → 132a (0.993)
16	2.414	19471	131a → 132a (0.714)	130a → 132a (0.713), 122b → 128b (0.266)
17	2.426	19565	126b → 127b (0.702)	122b → 128b (0.695), 130a → 132a (0.272)
18	2.452	19779	126b → 127b (0.944)	122b → 129b (0.926)
19	2.517	20298	126b → 127b (0.950)	121b → 129b (0.556), 121b → 128b (0.383)
20	2.529	20398	126b → 127b (0.937)	121b → 128b (0.526), 121b → 129b (0.392)

Notes: a = alpha (spin-up), b = beta (spin-down) orbitals. NTO occupations below 0.01 are omitted. Only single-excitation weights > 0.1 are shown. For State 12, the three dominant NTO occupations are 0.456, 0.286, and 0.222.
